# Transcriptomic evidence for a trade‐off between germline proliferation and immunity in *Drosophila*

**DOI:** 10.1002/evl3.261

**Published:** 2021-10-21

**Authors:** Marisa A. Rodrigues, Antoine Merckelbach, Esra Durmaz, Envel Kerdaffrec, Thomas Flatt

**Affiliations:** ^1^ Department of Biology University of Fribourg CH‐1700 Fribourg Switzerland

**Keywords:** Costs of reproduction, gene expression, germline, immunity, trade‐offs

## Abstract

Life‐history theory posits that investment into reproduction might occur at the expense of investment into somatic maintenance, including immune function. If so, reduced or curtailed reproductive effort might be expected to increase immunity. In support of this notion, work in *Caenorhabditis elegans* has shown that worms lacking a germline exhibit improved immunity, but whether the antagonistic relation between germline proliferation and immunity also holds for other organisms is less well understood. Here, we report that transgenic ablation of germ cells in late development or early adulthood in *Drosophila melanogaster* causes elevated baseline expression and increased induction of Toll and Imd immune genes upon bacterial infection, as compared to fertile flies with an intact germline. We also identify immune genes whose expression after infection differs between fertile and germline‐less flies in a manner that is conditional on their mating status. We conclude that germline activity strongly impedes the expression and inducibility of immune genes and that this physiological trade‐off might be evolutionarily conserved.

**Impact Summary**

A fundamental tenet of life‐history theory and evolutionary theories of aging is the existence of a trade‐off between germline activity (or reproduction more broadly) and somatic maintenance (and hence survival). A major physiological system that is thought to underpin this trade‐off, at least in part, is the immune system. Here, we confirm this prediction by demonstrating that removal of a proliferating germline in *Drosophila melanogaster*—relative to fertile flies with an intact germline—causes elevated baseline expression of immune genes and increases their induction upon infection. These findings thus provide direct experimental evidence that germline proliferation incurs an immunity cost of reproduction at the transcriptional level. Together with similar recent observations in germline‐ablated nematode worms, our results suggest that the trade‐off between germline activity and immune function might be evolutionarily conserved. Determining the molecular mechanisms underlying this reproduction‐immunity trade‐off is an important goal for future work.

A central tenet of life‐history theory is the existence of costs of reproduction, that is, trade‐offs between reproduction and other fitness components such as survival (Williams 1966; Calow 1979; Bell and Koufopanou 1986; Stearns 1989; Rose and Bradley 1998; Zera and Harshman 2001; Stearns and Magwene 2003; Harshman and Zera 2007; Flatt 2011; Flatt and Heyland 2011; Chen et al. 2020; Flatt 2020). Such trade‐offs are typically thought to arise from the competing energetic demands of reproduction versus those of other fitness traits; however, in principle, they might also be due to signaling processes independent of resource allocation (Leroi 2001; Barnes and Partridge 2003; Harshman and Zera 2007; Flatt 2011, 2020).

A major physiological system that might underpin trade‐offs between reproduction and survival is the immune system (Sheldon and Verhulst 1996; Lochmiller and Deerenberg 2000; Fedorka et al. 2004; Graham et al. 2011; Schwenke et al. 2016; Naim et al. 2020). Indeed, a large body of work has documented trade‐offs between reproductive processes (including mating) and immune function, both in invertebrates and vertebrates (Sorci et al. 1996; Norris and Evans 2000; Adamo et al. 2001; McKean and Nunney 2001; Fedorka et al. 2004; Greenman et al. 2005; Schmid‐Hempel 2005; Williams 2005; French et al. 2007; Martin et al. 2008; Miyata et al. 2008; Speakman 2008; Schwenke et al. 2016; Nystrand and Dowling 2020; Pick et al. 2020).

Despite this evidence, many fundamental aspects of reproduction‐immunity trade‐offs remain poorly understood (Rolff and Siva‐Jothy 2002; Flatt et al. 2005; Harshman and Zera 2007; Flatt et al. 2008a; Speakman 2008; Schwenke et al. 2016; Schwenke and Lazzaro 2017; Fabian et al. 2018; Garschall and Flatt 2018; Naim et al. 2020; Gupta et al. 2021). This not only includes specific questions about underlying molecular or physiological mechanisms, but also more basic questions, including the issue of whether such trade‐offs are symmetrical or not. For example, much work has demonstrated that immune activation compromises reproductive output (Zerofsky et al. 2005; reviewed in Schwenke et al. 2016 and Nystrand and Dowling 2020), but the flipside—that is, the question of whether reduced reproduction promotes immunity—has rarely been investigated. If immune suppression mediates the trade‐off between increased reproductive effort and decreased survival, we expect that abolished reproduction might promote immunity—as well as life span (Fedorka et al. 2004), or somatic maintenance more generally (Maklakov and Immler 2016; Chen et al. 2020).

This issue has been most thoroughly explored in the nematode *Caenorhabditis elegans* (and in related *Pristionchus* species) where germline removal (or sterility more generally) increases innate immunity, including improved resistance to bacterial infection (Miyata et al. 2008; Alper et al. 2010; Tekippe and Aballay 2010; Rae et al. 2012; Sinha and Rae 2014; Yunger et al. 2017). Germline ablation in *C. elegans* and *Pristionchus* also promotes longevity by activating the transcription factor DAF‐16/FOXO repressed by insulin/insulin‐like growth factor signaling (IIS) (Hsin and Kenyon 1999; Arantes‐Oliveira et al. 2002; Rae et al. 2012). Overall, these results are in good agreement with the hypothesis that survival costs of reproduction might be mediated by immune suppression (Fedorka et al. 2004). However, whether germline removal also promotes immune function in more distantly related animals is less clear.

Previous work by Short and collaborators (2012) has begun to tackle this question in *Drosophila melanogaster* by using a germline‐less mutant, *tudor*, which fails to form a primordial germline during embryonic development. Intriguingly, although mating reduced immune function (survival upon infection) of fertile wild‐type females, mated mutant females lacking germ cells did not suffer from decreased survival after infection (Short et al. 2012). Thus, the immune response to infection in mated flies appears to be germline dependent. By contrast, subsequent microarray analyses suggested that the general transcriptomic response to infection might not be germline dependent: qualitatively similar to fertile females, eggless *tudor* females upregulated the expression of many canonical immunity genes upon infection (Short and Lazzaro 2013). Potential expression differences between germline‐less and fertile flies in response to infection were, however, not formally tested.

Here, we have sought to revisit the question of germline dependence in *D. melanogaster* by using an alternative method for germline ablation. Because *grandchildless*‐like mutants such as *tudor* act during development (Boswell and Mahowald 1985), their effect on adult immunity might involve confounding pleiotropic side effects. Perhaps due to such effects, germline‐less *tudor* females are not long‐lived as adults (Barnes et al. 2006; Flatt et al. 2008b), in contrast to *C. elegans* and *Pristionchus* where germline removal robustly extends life span. We therefore used a transgenic system that eliminates germ cells (and thus abolishes fecundity) in *D. melanogaster* exclusively in late development or the adult stage, an intervention that has previously been reported to extend life span by modulating the activity of the IIS pathway (Flatt et al. 2008b). We combined this manipulation of germline activity with manipulations of infection and mating status and then employed RNA‐sequencing (RNA‐seq) to study transcriptome‐wide gene expression changes in response to these treatments. The principal objective of our transcriptomic analyses was to examine potential “conflicts” (trade‐offs) over patterns of gene expression between germline proliferation and the immune system (Stearns and Magwene 2003). Our findings show that germline ablation in *D. melanogaster* causes elevated baseline expression of immunity genes and increases their induction upon infection.

## Results and Discussion

To investigate immunity costs of reproduction at the gene expression level in *D. melanogaster*, we manipulated germline proliferation and mating status of female flies (see Supporting Information for details), following a similar experimental design as used by Short and Lazzaro (2013). To abolish germline proliferation (and hence fecundity), we drove overexpression of *bag of marbles* (UASp‐*bam* ^+^) with a germline‐specific *nanos* (*nos*)‐GAL4::VP16 driver, causing loss of germ cells in the late L3 and adult stage (Chen and McKearin 2003; Flatt et al. 2008b). Using RNA‐seq, we profiled whole‐body gene expression changes in sterile flies, as well as in two fertile control genotypes, in response to (a) infection with the gram‐negative bacterium *Pectobacterium (Erwinia) carotovora carotovora* (*Ecc15*) or the gram‐positive bacterium *Enterococcus faecalis* (*Ef*) 3 h post infection (relative to aseptic injury [pricking] controls, controlling for the confounding effects of wounding), and (b) mating (mated vs. virgin females), using a factorial design (see Supporting Information). We used two different bacteria to activate both the Imd and Toll innate immune pathways. The Imd pathway responds to infections with gram‐negative bacteria, whereas the Toll pathway is activated by infections with gram‐positive bacteria (e.g., De Gregorio et al. 2002; Leulier et al. 2003; Lemaitre and Hoffmann 2007; Sackton et al. 2010); however, some cross talk between these pathways has been documented (e.g., Leulier et al. 2000; Tzou et al. 2000; Tanji et al. 2007).

**Figure 1:**
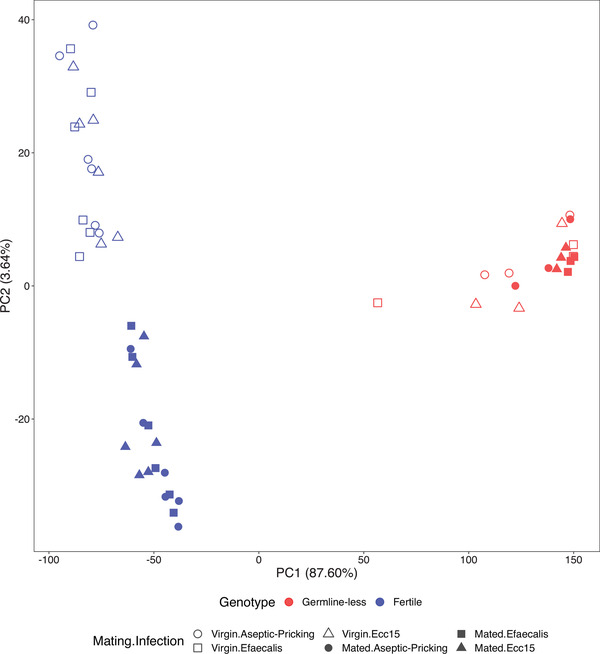
Principal component analysis (PCA) of differentially expressed genes (DEG). PC1 separates fertile and germline‐less flies, whereas PC2 separates virgin versus mated individuals within the group of fertile control flies, but not in the group of germline‐less flies. Germline removal clearly has a major effect on gene expression; other factors tend to have more subtle effects. The different colors represent the germline (fecundity) manipulation (red: germline‐less, sterile flies; blue: fertile control flies with intact germline); open versus filled symbols represent the mating treatment (open symbols: virgin females; filled symbols: mated females); and the different symbol shapes represent the infection treatments (circles: aseptic prick controls; triangles: infection with *Ecc15*; squares: infection with *Ef*).

In total, we identified 9169 differentially expressed genes (DEG) (Table S1). When comparing expression differences between germline‐less flies and either of the two control genotypes separately, we found that 98% of the DEG were identical in the two germline‐less versus control comparisons; we thus pooled data from both control genotypes for analysis (see Supporting Information). We first used principal component analysis (PCA) to explore overall patterns of gene expression differences (Fig. 1).

The first principal component (PC1) separated fertile and germline‐ablated flies into two markedly distinct clusters, explaining ∼88% of the variance and suggesting a major effect of germline proliferation versus removal on patterns of expression (Fig. 1; Table S1; also see Table S2). PC2 only explained ∼4% of the variance and separated mated versus virgin samples within the group of fertile control flies, but interestingly no such separation was apparent for sterile flies (Fig. 1; Table S1; also see Table S2). Thus, mating had a strong effect on gene expression in fertile but not in germline‐less flies, indicating the existence of an interaction between the state of germline activity and mating status, as previously observed by Short and Lazzaro (2013) (Table S2; also see results and discussion below). The first two PCs did not result in a clear separation of the three infection treatment groups (aseptic prick control vs. prick infection with *Ecc15* or with *Ef*) (Fig. 1), although many genes changed their expression in response to infection (Table S1; cf. Table S2). The lack of a clear separation of the infection groups might be explained by the fact that we measured expression 3 h after infection, when the immune system is in its early stages of activation, and because control flies were wounded (aseptic injury, i.e., to control for the effects of pricking), which is sufficient to elicit a weak immune response (Lemaitre et al. 1997).

We next systematically analyzed expression differences between experimental groups using linear models implemented in Limma‐Voom (Ritchie et al. 2015; see Supporting Information). To be conservative, we restricted all analyses (including pathway enrichment and gene ontology [GO] analyses) to statistically significantly DEG with an absolute fold change (FC) ≥ 2 (log_2_ [FC = 2] ≤ −1 or log_2_ [FC = 2] ≥ 1) (see Tables S1 and S2; see Supporting Information).

### GERMLINE PROLIFERATION TRADES OFF WITH IMMUNE GENE EXPRESSION

In the light of potential trade‐offs between germline proliferation and immunity, we aimed to identify effects on gene expression of (i) reproduction (R; germline‐less vs. fertile females) and (ii) the interaction between reproduction (R) and infection (I; aseptic prick control vs. infection, separately for each pathogen) (i.e., R × I interaction). In this context, we also analyzed the main effects of infection (I) on expression. Although we performed analyses transcriptome‐wide, we focused on investigating expression changes in immunity genes (a discussion of other expression changes is beyond the scope of this article).

At the transcriptome‐wide level, we identified 258 and 139 DEG affected by infection with *Ecc15* and *Ef*, respectively (Table S1). We generally found a larger number of DEG for flies infected with *Ecc15* than with *Ef* (Table S1). This might be due to our usage of a higher infection dose for *Ecc15*, as this strain is not pathogenic for its host but can induce an immune response (see Supporting Information; also see Basset et al. 2000, 2003). A not mutually exclusive alternative is that the Imd pathway, which is activated by *Ecc15*, might be more strongly induced than the Toll pathway; however, we did not find strong support for differences in the strength of induction between the two pathways (see Supporting Information).

**Figure 2:**
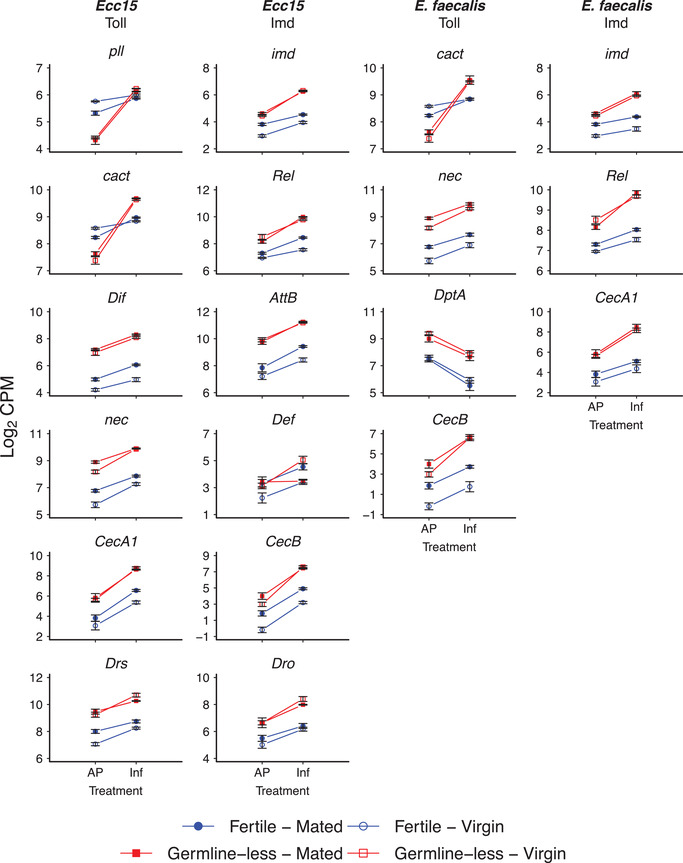
Infection with *Ecc15* and *Ef* induces a robust transcriptional immune response. The figure displays a selection of immune genes affected by the statistical main effect of bacterial infection (also see Tables S1 and S2 and Supporting Information). The first and second columns show DEG for flies infected with *Ecc15* (left column: DEG belonging to the Toll pathway; right column: DEG in the Imd pathway). The third and fourth columns show DEG for flies infected with *Ef* (left column: DEG belonging to the Toll pathway; right column: DEG in the Imd pathway). The *x*‐axes display the different infection treatments (AP: aseptic prick injury control; Inf: bacterial prick infection); the *y*‐axes show the log_2_ of the counts per million (CPM) values for a given gene. Germline‐less (sterile) flies are shown in red, and fertile control flies in blue; open symbols represent unmated virgin females, whereas filled colored symbols represent mated female flies. Error bars represent standard errors of the mean. Note that the expression patterns of *pll*, *imd*, *cact*, *Rel*, *Dif*, *nec*, *Def*, *CecB*, and *Dro* displayed above are also shown in Figure 3 and/or Figure 4 because the expression of these genes was also affected by the main effect of reproduction (R) and/or by the R × I interaction, respectively. Also see Figure 1 and Tables S1–S4.

As expected, DEG were enriched for pathways and GO terms related to immunity, especially for flies infected with *Ecc15* (Tables S3 and S4). Indeed, many DEG represent well‐known, canonical immunity genes (Fig. 2; Table S2) whose expression is well known to respond to infection (Basset et al. 2000; De Gregorio et al. 2002; Rutschmann et al. 2002; Buchon et al. 2009; Sackton et al. 2010).

Figure 2 shows examples of immunity genes whose expression was significantly affected by the main effect of infection (also see Table S1; for a full list, see Table S2). Many of these DEG represent members of the Toll and Imd innate immune signaling pathways (for background on these pathways, see Lemaitre et al. 1997; De Gregorio et al. 2002; Rutschmann et al. 2002; Lemaitre and Hoffmann 2007; Kleino and Silverman 2014). As expected from previous work, most of these genes were upregulated in response to infection (Fig. 2). These include major Imd and Toll signaling components, for example, the adapter protein *imd* (*immune deficiency*; [FlyBase gene number] FBgn0013983; [gene annotation ID] CG5576); the NF‐κB transcription factors *Dif* (*Dorsal‐related immunity factor*; FBgn0011274; CG6794) and *Rel* (*Relish*; FBgn0014018; CG11992); and the Toll antagonist *cact* (*cactus*; FBgn0000250; CG5848; induction of antagonists is common upon pathway activation—see below). We also observed infection‐induced upregulation of antimicrobial peptides (AMPs), the downstream targets of Toll and Imd signaling, including *AttB* (*Attacin‐B*; FBgn0041581; CG18372); *Def* (*Defensin*; FBgn0010385; CG1385); *CecA1* (*Cecropin A1*; FBgn0000276; CG1365) and *CecB* (*Cecropin B*; FBgn0000278; CG1878); *Drs* (*Drosomycin*; FBgn0283461; CG10810); and *Dro* (*Drosocin*; FBgn0010388; CG10816) (Fig. 2).

As can be seen from the “reaction norms” in Figure 2, many immune genes seem to exhibit qualitatively similar (i.e., parallel) expression responses to infection status between fertile and germline‐less females, consistent with the suggestion by Short and Lazzaro (2013) that the response to infection might not be germline dependent. However, closer inspection of the data in Figure 2 suggests that there might also exist differences in the expression profiles between germline‐less and fecund females; to address this issue, we now turn to analyzing the main and interaction effects of reproduction.

Linear models revealed that germline removal had major transcriptome‐wide effects on gene expression, confirming the PCA results in Figure 1. In total, ∼72% of all DEG were affected by the main effect of reproduction (see Table 2 for a full list of DEG; also see Fig. 1 and Table S1). Reproduction (i.e., germline‐less vs. fertile females) affected numerous fundamental biological functions, as reflected in significant enrichment of pathways and GO terms, including processes such as cell cycle regulation, development, and DNA and RNA metabolism (Tables S5 and S6).

**Figure 3:**
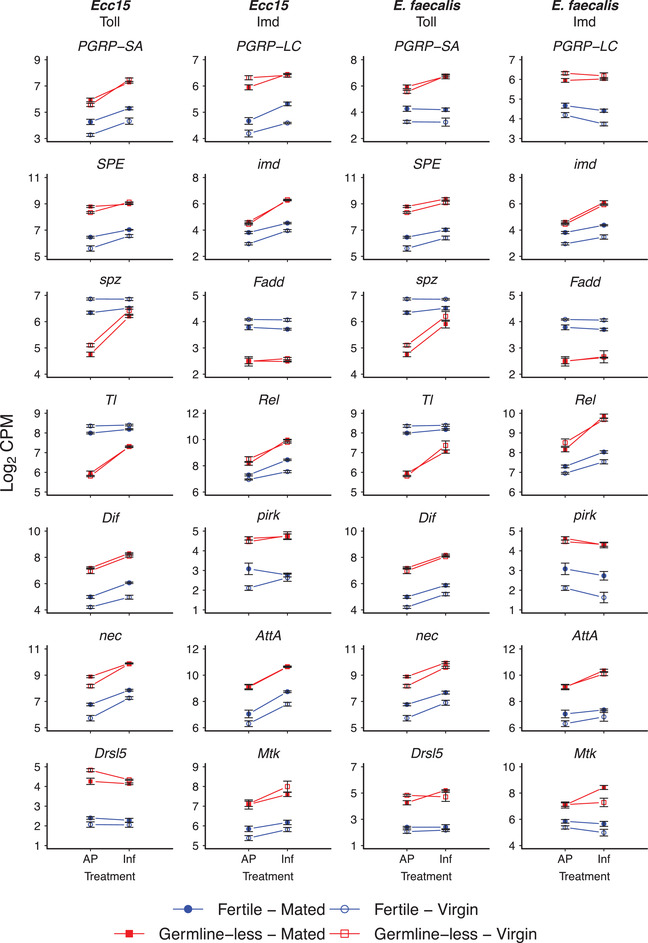
Germline removal causes constitutively higher immune gene expression. The figure shows a selection of canonical immune genes belonging to the Toll and Imd pathways and whose expression is affected by the statistical main effect of “reproduction” ([R]; germline loss vs. normal fecundity). For a full list of genes affected by reproduction, see Table S2; also see the results in Figure 2. The first and second columns show DEG for flies infected with *Ecc15* (left column: DEG belonging to the Toll pathway; right column: DEG in the Imd pathway). The third and fourth columns show DEG for flies infected with *Ef* (left column: DEG belonging to the Toll pathway; right column: DEG in the Imd pathway). The *x*‐axes display the different infection treatments (AP: aseptic prick injury control; Inf: bacterial prick infection); the *y*‐axes show the log_2_ of the counts per million (CPM) values for a given gene. Germline‐less (sterile) flies are shown in red, and fertile control flies in blue; open symbols represent unmated virgin females, whereas filled colored symbols represent mated female flies. Error bars represent standard errors of the mean. Note that the same data for *PGRP‐SA*, *imd*, *spz*, *Tl*, *Rel*, *Dif*, *nec*, and *Mtk* are also shown in Figure 2 and/or Figure 4 because their expression was also affected by the main effect of infection (I) and/or the R × I interaction, respectively.

In particular, the presence versus absence of a proliferating germline had a strong impact on the expression of many immune genes, including genes belonging to the Toll and Imd innate immune pathways, the melanization pathway, and the *Turandot* family (Table S2). Figure 3 shows a selection of immune genes whose expression was affected by germline loss versus normal fecundity (see Table S2 for full results; also see the results in Fig. 2).

Many immune genes showed constitutively higher expression in germline‐less flies as compared to fertile flies, independent of infection status (Fig. 3; Table S2): for example, the peptidoglycan recognition proteins *PGRP‐SA* (FBgn0030310; CG11709) and *PGRP‐LC* (FBgn0035976; CG4432); the signaling components *imd*, *Dif*, and *Rel*; and the AMP genes *AttA* (*Attacin‐A*; FBgn0012042; CG10146), *Drsl5* (*Drosomycin‐like 5*; FBgn0035434; CG10812), and *Mtk* (*Metchnikowin*; FBgn0014865; CG8175).

Three negative regulators of Toll and Imd signaling, namely, *nec* (*necrotic*; FBgn0002930; CG1857), *pirk* (*poor Imd response upon knock‐in*; FBgn0034647; CG15678), and *PGRP‐LB* (FBgn0037906; CG14704; expression profile not shown; see Table S2) (Levashina et al. 1999; Zaidman‐Rémy et al. 2006; Kleino et al. 2008; Paredes et al. 2011), also showed increased expression in germline‐less flies relative to fertile control flies (Fig. 3). Elevated expression of such negative regulators, which exert negative feedback control, is a common feature of increased immune pathway activity upon infection (Aggarwal et al. 2008; Kleino et al. 2008; Paredes et al. 2011). Perhaps as a result of such feedback regulation, we saw lower expression of, for example, the *Toll* receptor gene (*Tl*; FBgn0262473; CG5490) and the Toll ligand *spätzle* (*spz*; FBgn0003495; CG6134) in germline‐less flies. (Lower expression could also be due to a lack of maternal deposition of developmentally important Toll transcripts into eggs because germline‐less females do not produce oocytes; however, we did not find evidence in support of this hypothesis [see Supporting Information].) Germline proliferation thus seems to impede the constitutive baseline expression of several immune genes, independent of infection status (also see Fig. 2).

Next, we examined whether germline‐less versus fertile flies differ in their expression response to infection (R × I interaction). For flies infected with *Ecc15* or *Ef* (relative to prick controls), we found a transcriptome‐wide total of 136 and 58 genes, respectively, whose expression change in response to infection differed between germline‐less and fecund flies (Table S1; see Table S2 for full list). Pathway and GO term analyses revealed that these “interaction” genes were, as expected, enriched for immunity‐related pathways and GO terms; some metabolic functions were overrepresented as well, such as lipid, carbohydrate, and amino acid metabolism (Tables S7 and S8).

**Figure 4:**
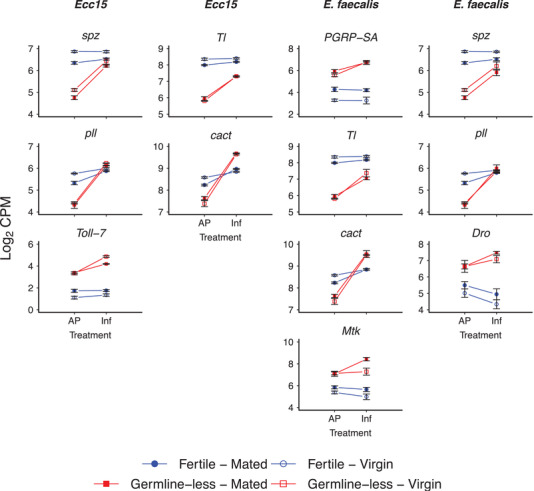
Induction of some immune genes by infection is germline dependent. The figure shows a small number of immune genes whose expression is affected by the statistical interaction between “reproduction” ([R]; (germline loss vs. normal fecundity) and “infection” ([I]; aseptic prick controls vs. infection with either *Ecc15* or *Ef*) (R × I interaction); the inducibility of these genes by infection is thus contingent upon the presence or absence of a proliferating germline. A full list of genes affected by this interaction is given in Table S2; also see the results in Figures 2 and 3. The first and second columns show DEG for flies infected with *Ecc15*; the third and fourth columns show DEG for flies infected with *Ef*. The *x*‐axes display the different infection treatments (AP: aseptic prick injury control; Inf: bacterial prick infection); the *y*‐axes show the log_2_ of the counts per million (CPM) values for a given gene. Germline‐less (sterile) flies are shown in red, and fertile control flies in blue; open symbols represent unmated virgin females, whereas filled colored symbols represent mated female flies. Error bars represent standard errors of the mean. Note that the same results for *spz*, *Tl*, *pll*, *cact*, *PGRP‐SA*, *Dro*, and *Mtk* are also displayed in Figure 2 and/or Figure 3 because their expression was also affected by the main effects of infection (I) and/or reproduction (R), respectively.

A few Toll pathway genes whose expression response was affected by the interaction between reproduction and infection are shown in Figure 4. Interestingly, for these genes, induction of expression upon infection was markedly higher in germline‐less females than in fertile females with an intact germline. Proliferation of germ cells thus not only impedes the constitutive baseline expression of many immune genes independent of infection status (see Fig. 3) but can also impact their inducibility upon infection (Fig. 4).

The effects of germline removal on immune gene expression might have functional consequences for the survival of flies after infection: using a survival assay of flies infected with *Ecc15*, we found that mated germline‐less females survive infection better than mated fertile females (see Supporting Information; Fig. S1), qualitatively consistent with similar observations by Short et al. (2012) using the germline‐less *tudor* mutant.

Our findings in *Drosophila* agree well with previous results from the nematode worm *Pristionchus pacificus* where germline ablation also induces constitutive upregulation of various immune genes (Rae et al. 2012)—this strongly suggests that the trade‐off between germline proliferation and immunity might be evolutionarily conserved.

### MATING EFFECTS ON IMMUNITY DEPEND ON GERMLINE PROLIFERATION

Another major reproductive process besides egg production is mating. Female flies are well known to undergo profound physiological changes in response to mating, including stimulation of egg production and oviposition (Kubli 2003; Kubli and Bopp 2012; Schwenke et al. 2016; Schwenke and Lazzaro 2017). Mating can also negatively impact immune function, especially survival after infection, in *Drosophila* and a variety of other insects (McKean and Nunney 2001; Rolff and Siva‐Jothy 2002; Fedorka et al. 2004, 2007; McGraw et al. 2004; Peng et al. 2005; Lawniczak et al. 2007; Short and Lazzaro 2010; Short et al. 2012; Schwenke et al. 2016).

Interestingly, Short et al. (2012) observed that the negative effect of mating upon survival after infection typically seen in fecund females is abolished in germline‐less flies: germline‐less mated females survived infection equally well as unmated fertile females and unmated germline‐less females, and all three groups survived infection substantially better than mated fertile females (also see below). Similarly, Short and Lazzaro (2013) found that the expression of three *Turandot* genes after infection depends on the interplay between reproductive status and mating status: in fertile flies, infection led to increased expression in virgin and mated females, whereas in germline‐less flies, expression was increased in virgin but not mated females. These observations indicate that infection‐induced immune responses to mating might be contingent on the state of germline activity. We were thus interested in exploring our transcriptomic dataset with regard to such germline dependent effects of mating on the expression of immune genes. Our results echo those of Short and Lazzaro (2013).

Interactions between the effects of reproduction and mating on patterns of gene expression were pervasive in our dataset. As can been seen from the transcriptome‐wide PCAs in Figure 1 above, PC2 separated virgin and mated flies into distinct clusters for fertile females but not for germline‐less females (also see Table S1). Linear models examining the main effects of mating (M) and the interactions between mating and infection (M × I) and between reproduction and mating (R × M) confirmed this pattern. At the whole‐transcriptome level, we identified 168 and 251 DEG whose expression levels were affected by mating in flies infected with either *Ecc15* and *Ef*, respectively, and 74 DEG that were affected by the M × I interaction in flies infected with *Ecc15* (Table S2; for enrichment analyses, see Table S9–S12). By contrast, a markedly larger number of genes were affected by the interaction between reproduction and mating (352 and 438 DEG in flies infected with *Ecc15* and *E. faecalis*, respectively; Table S2; also see Table S1; for enrichment analyses, see Tables S13 and S14). Notably, “interaction” genes were significantly enriched for two immunity‐related GO terms (“Antibacterial humoral response” and “Defense response to gram‐positive bacterium”) and mostly belonged to the Toll pathway (Table S14).

**Figure 5:**
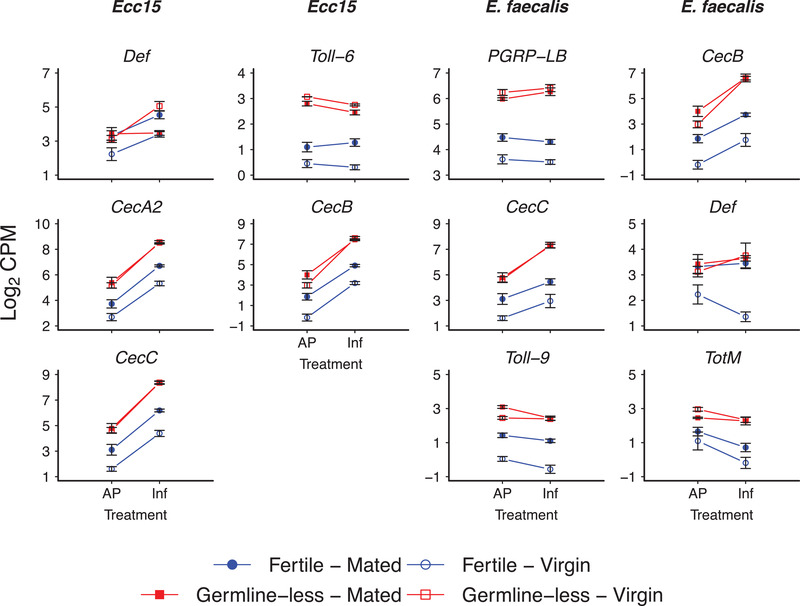
Mating effects on immune gene expression depend on germline proliferation. The figure shows a selection of immune genes affected by the R × M interaction (for the full list of genes affected by this interaction, see Table S2). The first and second columns show DEG for flies infected with *Ecc15*; the third and fourth columns show DEG for flies infected with *Ef*. The *x*‐axes display the different infection treatments (AP: aseptic prick injury control; Inf: bacterial prick infection); the *y*‐axes show the log_2_ of the counts per million (CPM) values for a given gene. Germline‐less (sterile) flies are shown in red, and fertile control flies in blue; open symbols represent unmated virgin females, whereas filled colored symbols represent mated female flies. Error bars represent standard errors of the mean. Note that *CecB* is also displayed in Figure 2 because its expression is also affected by the statistical main effect of infection (I).

Figure 5 displays immunity genes affected by the interaction between reproduction and mating (for a full list, see Table S2), including *PGRP‐LB*; two Toll‐like receptor family genes, *Toll‐6* (FBgn0036494; CG7250) and *Toll‐9* (FBgn0036978; CG5528); *Turandot M* (*TotM*; FBgn0031701; CG14027); and the AMPs *Def*, *CecA2*, *CecB*, and *CecC* (*Cecropin C*; FBgn0000279; CG1373). Although fertile mated females survive infections less well than fertile virgin females (Short et al. 2012; see Supporting Information; Fig. S1), many immune genes were upregulated in mated females as compared to virgin females with an intact germline (Fig. 5; also see results in Figs. 2, 3, 4). Although several studies have found that the magnitude of immune gene induction is smaller in mated as compared to virgin flies (Fedorka et al. 2007; Schwenke et al. 2016), increased expression is sometimes also observed, for reasons that are not entirely clear, and despite mating reducing survival after infection (cf. Short and Lazzaro 2013). Unlike the effects of mating seen in fertile flies, however, mating did not—or only very weakly—impact the expression of immune genes in germline‐less flies in our experiment (Fig. 5; also see results in Figs. 2, 3, 4).

These phenomenological observations thus suggest that the expression changes of several immune genes in response to mating activity are contingent upon the presence or absence of germline proliferation, as previously observed for some transcripts by Short and Lazzaro (2013). An interesting open question is whether such “interaction” genes might causally explain the improved survival of germline‐less flies after infection that, contrary to fertile flies, was found to be independent of mating status by Short et al. (2012). Similar to this study but using a different germline‐less genotype, we found that mated germline‐less female flies survived infection with *Ecc15* better than mated fertile females; yet, in contrast to Short et al. (2012), virgin fertile females survived infection better than virgin germline‐less females in our assay. In fact, mated germline‐less females survived approximately equally well as virgin fertile females, whereas mated fertile females survived approximately equally badly as virgin germline‐less females (see Supporting Information; Fig. S1). Together, the work of Short et al. (2012) and Short and Lazzaro (2013) as well as our experiments here reveal the existence of intricate interactions between germline proliferation (or lack thereof) and mating status that impact both immune gene expression and survival after infection.

What might be the likely physiological mechanisms that underpin the germline dependence of immune gene expression? Previous work has shown, for example, that downregulation of the IIS pathway can enhance survival of *D. melanogaster* after infection (Libert et al. 2008; McCormack et al. 2016), and suppression of immunity during reproduction in *C. elegans* depends on repression of the transcription factor DAF‐16/FOXO by IIS (Evans et al. 2008; Miyata et al. 2008; but see Alper et al. 2010). Because IIS is reduced in germline‐less flies (Flatt et al. 2008b), it might be an attractive possibility that the constitutive upregulation and increased inducibility of immune genes upon germline removal are caused by reduced IIS.

Working out the physiological regulation of the fecundity‐immunity trade‐off, and how it is modulated by mating, is a major goal for future research (cf. Schwenke et al. 2016). Important progress toward this end has recently been made by Schwenke and Lazzaro (2017). These authors found that, upon mating and transfer of male sex peptide contained in the seminal fluid, females upregulate the production of juvenile hormone (JH), a major gonadotropin with immunosuppressive effects (Flatt et al. 2008a), which severely reduces the resistance of flies to infections. These negative postmating effects on immunity could be experimentally rescued by ablation of the *corpus allatum* (CA) gland that produces JH (Schwenke and Lazzaro 2017). Interestingly, such CA‐ablated flies exhibit greatly reduced fecundity and increased life span, and JH synthesis is known to be regulated by IIS (see Yamamoto et al. 2013, and references therein). JH thus seems to represent a pleiotropic hormone involved in mediating—or modulating—trade‐offs between fecundity, immunity, and life span (Schwenke and Lazzaro 2017; reviewed in Flatt et al. 2005). It will clearly be very interesting to learn more about this and similar, yet to be identified mechanisms underlying the physiological regulation of reproductive trade‐offs.

## Conclusions

The fecundity‐immunity trade‐off represents a mutually antagonistic relationship. On the one hand, immune activation incurs a reproductive cost: female *D. melanogaster* exposed to heat‐killed bacteria lay significantly fewer eggs, but *imd* and *Rel* mutant females exhibit no such loss of fecundity (Zerofsky et al. 2005; Schwenke et al. 2016). On the other hand, reproduction incurs an immunity cost: mated germline‐less females survive infections much better than mated fertile females (Short et al. 2012; see Supporting Information; Fig. S1)—here, we have sought to identify transcriptional aspects of this immunity cost of reproduction.

Our experiments show that removal of the *Drosophila* germline in late development or early adulthood, as compared to female flies with an intact germline, causes (i) elevated constitutive expression of many immunity genes independent of infection status and (ii) stronger induction of some immune genes in response to bacterial infection. These results therefore reveal an immunity cost of reproduction at the transcriptional level that is attenuated upon germline loss. Together with similar findings in nematodes (Rae et al. 2012), these observations suggest that the effects of germline proliferation on immunity are evolutionarily conserved. Our transcriptomic data also corroborate previous results indicating that the immune response to mating is, in part, contingent upon germline proliferation (Short et al. 2012; Short and Lazzaro 2013). Although the detailed mechanisms await discovery, our results lend clear support to the fundamental idea that germline proliferation trades off with multiple aspects of somatic maintenance including immunity (Hsin and Kenyon 1999; Flatt et al. 2008b; Flatt 2011; Maklakov and Immler 2016; Chen et al. 2020).

## AUTHOR CONTRIBUTIONS

Definitions according to CRediT (https://casrai.org/credit/): MAR: Conceptualization, Data curation, Formal Analysis, Investigation, Methodology, Resources, Software, Validation, Visualization, Writing‐original draft, Writing‐review & editing; AM: Investigation, Methodology; ED: Methodology, Writing‐review & editing; EK: Software, Writing‐review & editing; TF: Conceptualization, Formal Analysis, Funding acquisition, Project administration, Supervision, Validation, Writing‐review & editing.

## DATA ARCHIVING

RNA‐seq data are available from the Short Read Archive (SRA) under SRA accession PRJNA721256 (https://www.ncbi.nlm.nih.gov/bioproject/721256).

## CONFLICT OF INTEREST

The authors declare no conflict of interest.

Associate Editor: R. Snook

## Supporting information


**Figure S1**. Reproduction has opposite effects on survival of mated versus virgin flies after infection with *Ecc15*.

**Table S1**. Total numbers of DEG in the different treatment groups.
**Table S2**. DEG identified using linear models.
**Table S3**. Pathways enriched for DEG affected by infection.
**Table S4**. GO terms enriched for DEG affected by infection.
**Table S5**. Pathways enriched for DEG affected by reproduction.
**Table S6**. GO terms enriched for DEG affected by reproduction.
**Table S7**. Pathways enriched for DEG affected by the interaction between reproduction and infection.
**Table S8**. GO terms enriched for DEG affected by the interaction between reproduction and infection.
**Table S9**. Pathways enriched for DEG affected by mating.
**Table S10**. GO terms enriched for DEG affected by mating.
**Table S11**. Pathways enriched for DEG affected by the interaction between mating and infection.
**Table S12**. GO terms enriched for DEG affected by the interaction between mating and infection.
**Table S13**. Pathways enriched for DEG affected by the interaction between reproduction and mating.
**Table S14**. GO terms enriched for DEG affected by the interaction between reproduction and mating.
**Table S15**. Experimental groups in the RNA‐seq experiment.
**Table S16**. Significant overlap in DEG when either of two fertile control genotypes is compared to the germline‐less genotype.
**Table S17**. Raw survival / mortality data from fly survival assay upon infection with *Ecc15*.
**Table S18**. Expression changes in Toll and Imd pathway genes in response to infection with *E. faecalis*.
**Table S19**. Relative strength of induction of the Toll vs. the Imd pathways upon infection with *E. faecalis*.
**Table S20**. Expression changes in Toll and Imd pathway genes in response to infection with *Ecc15*.
**Table S21**. Relative strength of induction of the Toll vs. the Imd pathways upon infection with *Ecc15*.
**Table S22**. Effect of germline removal on expression of immune genes that function either in immunity or in immunity and development.
