# Supplementary material for: Transcriptomic evidence for a trade‐off between germline proliferation and immunity in Drosophila
Source: Evol Lett. 2021 Oct 21;5(6):644–56. doi: 10.1002/evl3.261 (PMC8645197; doi:10.1002/evl3.261)
Supplement: Supplementary file 1 — Figure S1. Reproduction has opposite effects on survival of mated versus virgin flies after infection with Ecc15. [file EVL3-5-644-s001.docx]

***Supporting Information***

This supporting information file accompanies the paper by Rodrigues *et al.* (2021), “Transcriptomic Evidence for a Trade-off between Germline Proliferation and Immunity in *Drosophila*”.

**SUPPORTING MATERIALS AND METHODS**

***Drosophila* strains and maintenance**

To obtain germline-less flies, we used a binary GAL4>UAS system by crossing a *nanos*-GAL4::VP16 driver (full genotype: *yw; +/+; nanos-*GAL4::VP16*/nanos-*GAL4::VP16; Van Doren *et al.* 1998) to a UASp-*bag of marbles* (*bam*) responder line (*bam;* full genotype: *y.w;* UASp-*bam*^+^*/CyO*; +/+; Chen and McKearin 2003) (see Flatt *et al.* 2008b for further details). Ectopic overexpression of *bam* under the control of the *nanos*-GAL4::VP16 driver causes loss of germ cells at the late L3 pupal stage or in early adulthood (Chen and McKearin 2003; Flatt *et al*. 2008b). The above cross yields 50% germline-less flies (*y,w; nanos*-GAL4::VP16/+; UASp-*bam*/+) and 50% fertile flies (*y,w; CyO*/+; *nanos*-GAL4::VP16/+). As fertile control genotypes we used (i) the fertile females derived from this cross and (ii) fertile *y^1^, w^1118^* mutant females (*y*^1^,*w*^1118^; +/+; +/+), *i.e.* the strain that provided the genetic background for both the UAS and GAL4::VP16 strains (obtained via multi-generation backcrossing; see Flatt *et al*. 2008b). Flies were maintained and experiments performed at 25°C and 60% relative air humidity on a 12h:12h light:dark cycle, using controlled larval densities.

**Experimental flies**

Adult virgin females and males from the *nanos*-GAL4::VP16 and UASp-*bam* lines were collected within a 2-hour interval upon eclosion and kept separately prior to crosses. After three days, 15 females from one strain and 15 males from the other strain were placed together in a bottle for mating and egg laying during 24 hours. Crosses were set up in both directions (cross 1: virgin females of UASp-*bam* x virgin males of *nanos*-GAL4::VP16; cross 2: virgin females of *nanos*-GAL4::VP16 x virgin males of UASp-*bam*). From these crosses, we reared F1 progeny to adulthood under the above-mentioned conditions. Adult F1 flies were collected within 2 hours of eclosion and sexed under light CO_2_ anesthesia. Next, we separated females of each genotype (germline-less flies: *y,w; nanos-*GAL4::VP16/+; UASp-*bam*/+; and fertile control flies: *y,w*; *CyO*/+; *nanos*-GAL4::VP16/+) into two groups: females that would remain virgins and females that would mate with their male siblings. The second control (*y*^1^,*w*^1118^) was handled as described above for transgenic crosses. Flies were provided with fresh food every second day during four days prior to infection (see below). Preliminary assays revealed no differences in life-history between the two directions of the cross (unpublished data), so that we decided to pool adult offspring from both crosses in equal proportions for experiments.

**Bacterial infection of flies**

We used two bacterial strains for infection (courtesy of Bruno Lemaitre, EPFL, Lausanne): a Gram-positive bacterium (*Enterococcus faecalis*), which primarily triggers an immune response via the Toll pathway; and a Gram-negative bacterium (*Pectobacterium* [*Erwinia*] *carotovora carotovora*; also known as *Ecc15*), which primarily triggers an immune response via the Imd pathway (e.g., De Gregorio et al. 2002; Leulier *et al.* 2003; Lemaitre and Hoffmann 2007; **Sackton *et al.* 2010)**. However, these pathways are known to be subject to some cross-talk (e.g., Leulier *et al.* 2000; Tzou *et al.* 2000; Tanji *et al.* 2007). Bacteria were kept on culture plates at 4°C until the day prior to infecting flies. In the evening prior to infection, a single colony was aseptically picked from each bacterial plate and cultured overnight in liquid Luria/Miller (LB) broth (Roth #X968.2) at the optimal culture temperature (29°C for *Ecc15*; 37°C for *E. faecalis*) with continuous shaking. The next day, bacterial suspensions were centrifuged for 15 minutes at 3500 rpm, and the supernatant was discarded. Bacterial pellets were resuspended in LB medium until reaching a concentration of OD_600_ = 250 (+/- 5%) and OD_600_ = 5 (+/- 5%) for *Ecc15* and *E. faecalis*, respectively (Mark Hanson, pers. comm.). Because *Ecc15*, a phytopathogen which uses insects as a vector, is non-pathogenic to its *D. melanogaster* host but can trigger a systemic immune response (Basset *et al.* 2000, 2003; Nadarasah and Stavrinides 2011), we used a high concentration to induce a robust immune response. Since *E. faecalis* can cause high mortality of infected lab-reared fly strains (Lazzaro 2002; Cox and Gilmore 2007; Chandler *et al.* 2011), we used a lower bacteria concentration. For infections, 4-day-old females were anesthetized with CO_2_ and infected with a 0.1 mm diameter pin needle (Fine Scientific Tools #26002-10) dipped into bacterial pellet (Neyen *et al*. 2014). Flies were pricked on the left side of the thorax, below the wing. After pricking, flies were placed into vials with fly food (0.7% agar; 5% sugar; 5% dry yeast; 1% nipagin diluted to a 20% solution; 0.6% propionic acid) and transferred to 25ºC after recovery from anesthesia. Experimental flies (3 genotypes x 2 mating groups [virgin vs. mated]) were either infected with *Ecc15* or with *E. faecalis*, or aseptically pricked with a dry sterile needle to control for the effects of injury without infection. Injury due to aseptic pricking is sufficient to trigger the production of AMPs (Lemaitre *et al*. 1997). For each treatment group, we pricked 18 flies to generate 3 replicates per group, with 6 flies pooled per replicate (see Table S15). Approximately three hours after infection, females were snap-frozen in liquid nitrogen before being stored at -80°C until RNA extraction.

**RNA extraction and RNA-sequencing**

We extracted total RNA using a Maxwell® RSC simply RNA Tissue (AS1340) kit on a Maxwell® RSC instrument (Promega, AS4500), following the manufacturer's protocols. After extraction, we assessed RNA quality with a bioanalyzer (Agilent Technologies 2200 TapeStation; RNA ScreenTape Sample Buffer [5067-5577]; RNA ScreenTape Ladder [5067-5578]; RNA ScreenTape [5067-5576]). Total RNA was stored at -80°C prior to sequencing. RNA sequencing (RNA-seq) was performed at Eurofins Genomics Europe Sequencing (Germany), using the Illumina HiSeq 4000 platform (single-end reads, 50 bp read length, aiming for 30 million sequenced reads).

**Transcriptomic and statistical analyses of expression data**

After sequencing, we performed quality assessment using FastQC (v.0.11.8; Andrews 2010) and trimmed reads with Q-score of 20 using Cutadapt (v.1.15; Martin 2011). Trimmed reads were aligned to the *D. melanogaster* transcriptome (release 6.17) using topHat (v.2.1.0; Kim *et al*. 2013), and a quantification list of transcript abundances was generated using featureCounts (v.1.4.6-p5; Liao *et al*. 2014). To identify differentially expressed genes in our data we used the Bioconductor package edgeR (v.3.26.8; Robinson *et al*. 2009) in *R* (v.3.6.1; <http://www.R-project.org>). Genes with less than two counts per million in at least three samples were excluded. The final number of differentially expressed genes (DEG) in our dataset was 9169. We first analyzed both control genotypes to determine whether they differ from each other by comparing each of them separately to the germline-less genotype. Using the *R* package SuperExactTest (v.1.0.7; Wang *et al*. 2015), we found that 98% of the DEG identified in comparisons of either of the two control genotypes to the single germline-less genotype were identical and thus independent of the control genotype used (Table S16). We thus pooled the data from both control genotypes and proceeded with factorial analysis of pooled fertile control genotypes versus the single germline-less genotype. To carry out factorial analyses of treatment effects on gene expression we used the Bioconductor package Limma-Voom (v.3.40.6; Ritchie *et al*. 2015). For each bacterial pathogen separately, we sought to identify genes whose expression was significantly affected by each treatment (main effect): reproduction (R, germline-less versus fertile), infection (I, aseptic pricking versus bacterial infection), and mating status (M, virgin versus mated). We also analyzed the data with regard to potential two-way interactions, namely interactions between reproduction and mating (R x M), reproduction and infection (R x I), and mating and infection (M x I). For simplicity, we did not analyze potential 3-way interactions. To account for multiple testing, we applied the Benjamini-Hochberg correction to all *p*-values (Benjamini and Hochberg 1995). Next, we selected DEG with an adjusted *t*-test *p-value* < 0.05. Because some DEG exhibited rather small fold-changes (FC), we used an additional FC cutoff criterion and only considered genes with an absolute FC ≥ 2 as candidates for analysis (log_2_ [2] ≤ -1 or log_2_ [2] ≥ 1) (Table S1, Table S2). To perform pathway enrichment analyses, we used the Bioconductor package ReactomePA (v.1.28.0; Yu and He 2016). As a complementary approach, we also performed gene ontology (GO) term enrichment analyses using the Bioconductor package topGO (v2.36.0; Alexa and Rahnenführer 2010), with a minimum node size of five. To be conservative, we only considered terms with adjusted *p*-values < 0.005 as being significant.

**Assay of fly survival upon infection with *Ecc15***

To test whether reproduction affects survival upon infection, we measured post-infection survival of mated and virgin germline-less female flies (*y,w; nanos*-GAL4::VP16/+; UASp-*bam*/+) and of mated and virgin fertile female flies (*y,w; CyO*/+; *nanos*-GAL4::VP16/+) after infection with *Ecc15* (OD_600_ = 200 +/- 5%), following the general infection protocol described above. After pricking, flies were transferred to vials with normal fly food and vials were kept on their sides until flies had recovered from anesthesia. Flies that died within 6 hours after infection were excluded from analysis and considered dead by injury (Neyen *et al.* 2014). Flies were placed in groups of 10 females per vials, with 10 - 13 replicate vials for each of 4 treatment groups. Dead flies were scored every 12 hours. Surviving flies were transferred to new vials with fresh food every two days. Mortality data were analyzed with JMP (v.13.0.0), using a factorial Cox (proportional hazards) regression model with the factors ‘Reproduction’ (R; germline-less vs. fertile), ‘Mating’ (M; mated vs. virgin) and their interaction. In addition, to assess the significance of differences in survivorship between the two groups of interest (i.e., between germline-less and fertile flies) we performed post-hoc analysis with both log-rank and generalized Wilcoxon tests on mated and virgin flies, separately. We report both statistics because the former gives equal weight to all time points, whereas the latter gives more weight to deaths at early time points. The survival / mortality raw data are given in Table S17.

**Data availability**

RNA-seq data are available from the Short Read Archive (SRA): SRA accession: PRJNA721256 (<https://www.ncbi.nlm.nih.gov/bioproject/721256>).

**SUPPORTING RESULTS**

**Opposite effects of germline loss on infection survival in mated and virgin flies**

To examine whether reproduction affects survival upon infection, we measured post-infection survival of both mated and virgin germline-less and fertile female flies after infection with Ecc15 (see Table S17 for the raw data). Inspection of survivorship curves (see Fig. S1 below) and Cox regression analysis (see table below) revealed a significant reproduction-by-mating interaction effect on survival after infection with *Ecc15*. Post-hoc analysis of the interaction pattern revealed that mated germline-less females survive infection better than mated fertile females when using a generalized Wilcoxon test which gives more weight to early survival times (χ2 = 4.33, df = 1, *p* = 0.037), but not when using a log-rank test (χ2 = 3.19, df = 1, *p* = 0.074). Thus, germline proliferation in fertile flies seems to incur an immunity cost of reproduction in terms of survival after infection, consistent with previous observations by Short *et al.* (2012). By contrast, in virgin flies, fertile females survived infection better than germline-less females under both test statistics (generalized Wilcoxon test: χ2 = 6.45, df = 1, *p* = 0.011; log-rank test: χ2 = 5.99, df = 1, *p* = 0.014).

*
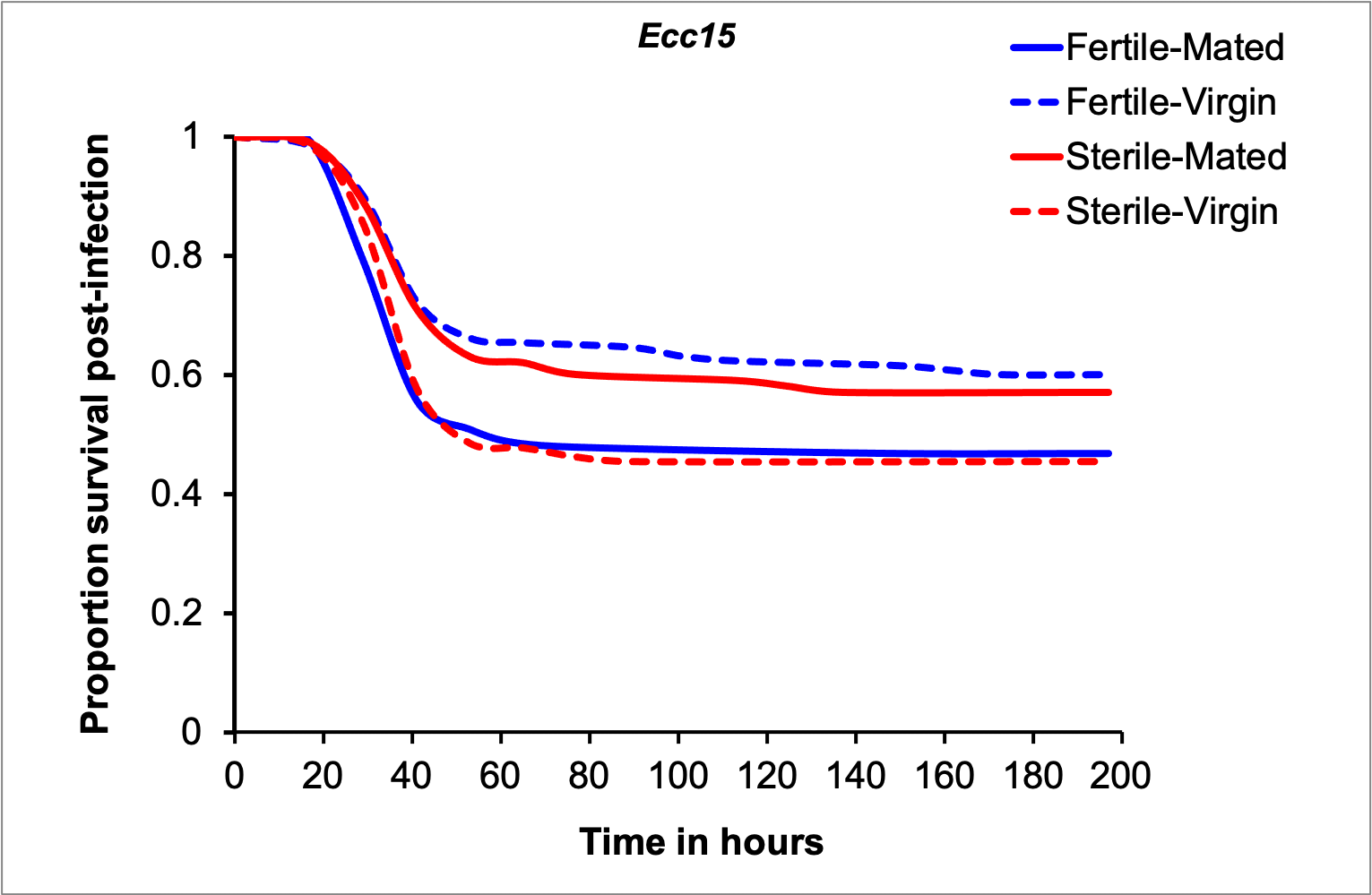
*

**Figure S1. Reproduction has opposite effects on survival of mated versus virgin flies after infection with *Ecc15*.** In mated females, germline-less flies survive infection better than fertile control flies, indicating the existence of an immunity cost of reproduction. In marked contrast, in infected virgin flies, the opposite pattern is seen: fertile virgin females survive better than germline-less virgin females. This interaction effect on post-infection survival is supported by a significant interaction between reproduction and mating in a Cox regression analysis (see table below). Interestingly, interaction effects between reproduction and mating are also observed for the expression of some immunity genes (see main text).

**Table: details of Cox regression analysis**

***Source df Wald χ2 Prob > χ2***

Genotype 1 0.08225582 0.7743

Mating 1 0.12515416 0.7235

Genotype x Mating 1 7.33319787 0.0068*

**Relative strength of induction of the Toll and Imd pathways upon infection**

To examine whether there might exist differences in the induction upon infection between the Toll and Imd pathways, we estimated the average level of induction upon infection for both pathways, averaging across all significantly infection-induced DEG in each pathway, and tested for differences in induction levels between the pathways by means of Welch *t*-tests (assuming unequal variances) (see Tables S18-S21 for the data and details of these calculations). Our quantification suggested that transcripts in the Imd pathway might be, on average, more strongly induced upon infection than those of the Toll pathway (Table S18, Table S20). Although this difference in induction strength between the pathways was significant in several individual cases, most test outcomes were not significant after Bonferroni correction (Table S19, Table S21). We conclude that, while it is possible that in our experiment the Imd pathway might have been more strongly induced by infection than the Toll pathway, there is no strong evidence in our data for such a difference in induction strength between the two pathways. It remains possible, however, that our statistical tests were underpowered, given the small number of genes in each pathway that entered our calculations.

**Expression of immunity genes that also have a developmental role**

For a few Toll genes we observed lower expression in germline-less relative to fertile flies after infection (e.g., see main text and Fig. 3), contrary to what we observed for the majority of Toll genes. This lower expression could potentially be due to a lack of maternal deposition of developmentally important Toll transcripts into eggs since germline-less females do not produce oocytes. We therefore classified the Toll (and Imd) pathway genes in our dataset into two categories: (1) those that are thought to play a role in immunity only, and (2) those that are thought to function both in immunity and development, using information from FlyBase (https://flybase.org/) and Duneau *et al*. (2017). Under the above hypothesis, one might expect that immunity genes with a function in both immunity and development would show lower expression in germline-less as compared to fertile flies, yet this would not be expected for immunity genes that function in immunity alone. While some genes with both immune and developmental function were indeed downregulated in germline-less flies, others were upregulated (Table S22). Overall, we therefore did not find clear support for the above hypothesis; in fact, the majority of Toll (and Imd) genes, including those with only immune function as well as those with both functions, were upregulated in germline-less flies as compared to fertile flies (Table S22).

**LITERATURE CITED**

Alexa, A., and J. Rahnenführer. 2010. topGO: Enrichment Analysis for Gene Ontology. Available online at: <https://bioconductor.org/packages/release/bioc/html/topGO.html>

Andrews S. (2010). FastQC: a quality control tool for high throughput sequence data. Available online at: <http://www.bioinformatics.babraham.ac.uk/projects/fastqc>

Basset, A., R. S. Khush, A. Braun, L. Gardan, F. Boccard, J. A. Hoffmann, *et al*. 2000. The phytopathogenic bacteria *Erwinia carotovora* infects *Drosophila* and activates an immune response. Proc. Natl. Acad. Sci. U. S. A. 97:3376-3381.

Basset, A., P. Tzou, B. Lemaitre, and F. Boccard. 2003. A single gene that promotes interaction of a phytopathogenic bacterium with its insect vector, Drosophila melanogaster. EMBO Rep. 4:205-209.

Benjamini, Y., and Y. Hochberg. 1995. Controlling the False Discovery Rate: A Practical and Powerful Approach to Multiple Testing. J. R. Stat. Soc. B 57:289–300.

Chandler, J. A., J. Lang, S. Bhatnagar, J. A. Eisen, and A. Kopp. 2011. Bacterial communities of diverse *Drosophila* species: Ecological context of a host-microbe model system. PLoS Genet. 7(9): e1002272.

Chen, D., and D.M. McKearin. 2003. A discrete transcriptional silencer in the *bam* gene determines asymmetric division of the *Drosophila* germline stem cell. Development 130:1159–1170.

Cox, C. R., and M. S. Gilmore. 2007. Native microbial colonization of *Drosophila melanogaster* and its use as a model of *Enterococcus faecalis* pathogenesis. Infect. Immun. 75:1565–1576.

De Gregorio, E., P. T. Spellman, P. Tzou, G. M. Rubin, and B. Lemaitre. 2002. The Toll and Imd pathways are the major regulators of the immune response in *Drosophila*. EMBO J. 21:2568–2579.

Duneau, D. F., H. C. Kondolf, J. H. Im, G. A. Ortiz, C. Chow, M. A. Fox, *et al*. 2017. The Toll pathway underlies host sexual dimorphism in resistance to both Gram-negative and Gram-positive bacteria in mated *Drosophila*. BMC Biol. 15:1–17.

Flatt, T., K.-J. Min, C. D’Alterio, E. Villa-Cuesta, J. Cumbers, R. Lehmann, *et al*. 2008b. *Drosophila* germline modulation of insulin signaling and lifespan. Proc. Natl. Acad. Sci. U. S. A. 105:6368–6373.

Kim, D., G. Pertea, C. Trapnell, H. Pimentel, R. Kelley, and S. L. Salzberg. 2013. TopHat2: accurate alignment of transcriptomes in the presence of insertions, deletions and gene fusions. Genome Biol. 14:1-13.

Lazzaro, B. P. 2002. A population and quantitative genetic analysis of the *Drosophila melanogaster* antibacterial immune response. Ph.D. Thesis, Pennsylvania State University.

Lemaitre, B., and J. Hoffmann. 2007. The Host Defense of *Drosophila melanogaster*. Annu. Rev. Immunol. 25:697–743.

Lemaitre, B., J. M. Reichhart, and J. A. Hoffmann. 1997. *Drosophila* host defense: Differential induction of antimicrobial peptide genes after infection by various classes of microorganisms. Proc. Natl. Acad. Sci. U. S. A. 94:14614–14619.

Leulier, F., C. Parquet, S. Pili-Floury, J. H. Ryu, M. Caroff, W. J. Lee, *et al*. 2003. The *Drosophila* immune system detects bacteria through specific peptidoglycan recognition. Nat. Immunol. 4:478–484.

Leulier, F., A. Rodriguez, R. S. Khush, J. M. Abrams, and B. Lemaitre. 2000. The *Drosophila* caspase Dredd is required to resist Gram-negative bacterial infection. EMBO Rep. 1:353–358.

Liao, Y., G. K. Smyth, and W. Shi. 2014. featureCounts: an efficient general purpose program for assigning sequence reads to genomic features. Bioinformatics 30:923–930.

Martin, M. 2011. Cutadapt removes adapter sequences from high-throughput sequencing reads. EMBnet.journal 17:10–12.

Nadarasah, G., and J. Stavrinides. 2011. Insects as alternative hosts for phytopathogenic bacteria. FEMS Microbiol. Rev. 35:555–575.

Neyen, C., A. J. Bretscher, O. Binggeli, and B. Lemaitre. 2014. Methods to study *Drosophila* immunity. Methods 68:116–128.

Ritchie, M. E., B. Phipson, D. Wu, Y. Hu, C. W. Law, W. Shi, *et al*. 2015. limma powers differential expression analyses for RNA-sequencing and microarray studies. Nucleic Acids Res. 43:e47.

Robinson, M. D., D. J. McCarthy, and G. K. Smyth. 2009. edgeR: A Bioconductor package for differential expression analysis of digital gene expression data. Bioinformatics 26:139–140.

Sackton, T. B., B. P. Lazzaro, and A. G. Clark. 2010. Genotype and gene expression associations with immune function in *Drosophila*. PLoS Genet. 6:e1000797.

Schmid-Hempel, P. 2005. Evolutionary ecology of insect immune defenses. Ann. Rev. Entomol. 50:529-551.

Short, S. M., M. F. Wolfner, and B. P. Lazzaro. 2012. Female *Drosophila melanogaster* suffer reduced defense against infection due to seminal fluid components. J. Insect Physiol. 58:1192–1201.

Tanji, T., X. Hu, A. N. R. Weber, and Y. T. Ip. 2007. Toll and IMD Pathways Synergistically Activate an Innate Immune Response in *Drosophila* *melanogaster*. Mol. Cell. Biol. 27:4578–4588.

Tzou, P., S. Ohresser, D. Ferrandon, M. Capovilla, J. M. Reichhart, B. Lemaitre, *et al*. 2000. Tissue-specific inducible expression of antimicrobial peptide genes in *Drosophila* surface epithelia. Immunity 13:737–748.

Van Doren, M., A.L. Williamson, and R. Lehmann. 1998. Regulation of zygotic gene expression in *Drosophila* primordial germ cells. Curr. Biol. 8:243–246.

Wang, M., Y. Zhao, and B. Zhang. 2015. Efficient Test and Visualization of Multi-Set Intersections. Sci. Rep. 5:1–12.

Yu, G., and Q. Y. He. 2016. ReactomePA: An R/Bioconductor package for reactome pathway analysis and visualization. Mol. Biosyst. 12:477–479.
